# Human microbiota drives hospital-associated antimicrobial resistance dissemination in the urban environment and mirrors patient case rates

**DOI:** 10.1186/s40168-022-01407-8

**Published:** 2022-12-02

**Authors:** Cecilia Salazar, Matias Giménez, Nadia Riera, Andrés Parada, Josefina Puig, Antonio Galiana, Fabio Grill, Mariela Vieytes, Christopher E. Mason, Verónica Antelo, Bruno D’Alessandro, Jimena Risso, Gregorio Iraola

**Affiliations:** 1 Microbial Genomics Laboratory, Institut Pasteur de Montevideo, 11400 Montevideo, Uruguay; 2 Molecular Microbiology Laboratory, Instituto de Investigaciones Biológicas Clemente Estable (IIBCE), Montevideo, Uruguay; 3 Hospital Maciel, Montevideo, Uruguay; 4 Department of Physiology and Biophysics, Weill Cornell Medicine, New York, NY USA; 5 The HRH Prince Alwaleed Bin Talal Bin Abdulaziz Alsaud Institute for Computational Biomedicine, Weill Cornell Medicine, New York, NY USA; 6 The WorldQuant Initiative for Quantitative Prediction, Weill Cornell Medicine, New York, NY USA; 7 Servicio de Evaluación de la Calidad y Control Ambiental, Intendencia de Montevideo, Montevideo, Uruguay; 8 Instituto de Higiene, Facultad de Medicina, Universidad de la República, Montevideo, Uruguay; 9 Wellcome Sanger Institute, Hinxton, UK; 10 Center for Integrative Biology, Universidad Mayor, Santiago de Chile, Chile

**Keywords:** Antimicrobial resistance, Urban metagenomics, Nanopore sequencing, Carbapenem resistance, Nosocomial outbreak, KPC, Urban wastewater

## Abstract

**Background:**

The microbial community composition of urban environments is primarily determined by human activity. The use of metagenomics to explore how microbial communities are shaped in a city provides a novel input that can improve decisions on public health measures, architectural design, and urban resilience. Of note, the sewage system in a city acts as a complex reservoir of bacteria, pharmaceuticals, and antimicrobial resistant (AMR) genes that can be an important source of epidemiological information. Hospital effluents are rich in patient-derived bacteria and can thus readily become a birthplace and hotspot reservoir for antibiotic resistant pathogens which are eventually incorporated into the environment. Yet, the scope to which nosocomial outbreaks impact the urban environment is still poorly understood.

**Results:**

In this work, we extensively show that different urban waters from creeks, beaches, sewage spillways and collector pipes enclose discrete microbial communities that are characterized by a differential degree of contamination and admixture with human-derived bacteria. The abundance of human bacteria correlates with the abundance of AMR genes in the environment, with beta-lactamases being the top-contributing class to distinguish low vs. highly-impacted urban environments. Indeed, the abundance of beta-lactamase resistance and carbapenem resistance determinants in the urban environment significantly increased in a 1-year period. This was in line with a pronounced increase of nosocomial carbapenem-resistant infections reported during the same period that was mainly driven by an outbreak-causing, carbapenemase-producing *Klebsiella pneumoniae* (KPC) ST-11 strain. Genome-resolved metagenomics of urban waters before and after this outbreak, coupled with high-resolution whole-genome sequencing, confirmed the dissemination of the ST-11 strain and a novel KPC megaplasmid from the hospital to the urban environment. City-wide analysis showed that geospatial dissemination of the KPC megaplasmid in the urban environment inversely depended on the sewage system infrastructure.

**Conclusions:**

We show how urban metagenomics and outbreak genomic surveillance can be coupled to generate relevant information for infection control, antibiotic stewardship, and pathogen epidemiology. Our results highlight the need to better characterize and understand how human-derived bacteria and antimicrobial resistance disseminate in the urban environment to incorporate this information in the development of effluent treatment infrastructure and public health policies.

Video Abstract

**Supplementary Information:**

The online version contains supplementary material available at 10.1186/s40168-022-01407-8.

## Introduction

Antimicrobial resistance (AMR) is defined as the process which allows bacteria to adapt and survive under the presence of normally inhibitory concentrations of antimicrobials. This process is of public health concern given that various prevailing human pathogens have evolved towards AMR phenotypes. Remarkably, the World Health Organization (WHO) has identified AMR as one of the most dangerous threats for human and food safety [1]. The problem has now exacerbated due to the COVID-19 pandemic that triggered a tremendous global increase of hospitalizations and antibiotic treatment within intensive care units [2]. Indeed, given the presence of immunocompromised patients and high antibiotic selective pressures, hospital environments are considered hotbeds for the emergence and dissemination of AMR [3]. In this context, *Klebsiella pneumoniae* has been identified as a major threat to human health causing nosocomial outbreaks mainly associated with multidrug resistance, including last-line antibiotics such as carbapenems around the world [4]. In addition to the clinical importance of this species, *K. pneumoniae* can be normally carried as part of the human gut microbiota [5] and survives in a wide range of host-associated and natural niches, making it an ideal vector for dissemination of AMR mechanisms from hospital settings to other environments [6].

A major problem for public health, particularly in low- and middle-income countries, is that hospital wastewater is contaminated with patient-derived microbiota, and this wastewater is directly incorporated into the urban environment through sewage systems without treatment. Given this complex sewage mixture, the use of whole-genome and metagenomic next-generation sequencing (NGS) approaches to analyze sewage waters for AMR surveillance is becoming increasingly applied. NGS-based methods constitute a non-invasive and straightforward strategy to gain insight on the overall health status of an urban population as a whole [7, 8]. Indeed, the global analysis of urban environments using metagenomics has revealed that they enclose a dynamic but established microbiome [9] and that urban sewage water systems represent an extensive reservoir of AMR [10]. This is relevant because the interaction of human-derived with environmental bacteria in the urban environment provides a suitable scenario for the spreading and emergence of AMR genes, since they are generally associated with mobile genetic elements like plasmids [11].

Here, we present a 2-year study of urban waters that combines metagenomics and long-read whole-genome sequencing of hospital-associated outbreak strains to characterize the city-scale circulation of AMR. Overall, we studied the effect that human contamination and hospital-associated outbreaks have on the dissemination of AMR in the urban environment, performed genome-resolved characterization of carbapenem-resistant clones transmitted from the hospital to urban waters, and revealed an effect of the built environment in the extensive dissemination of multidrug resistance plasmids. Together, our work demonstrates how high-resolution urban metagenomics and outbreak genomic epidemiology can be jointly applied to investigate the dynamics of AMR pathogens in the city environment.

## Results

### Study overview

Montevideo is the capital city of Uruguay, being situated along the Rio de la Plata estuary (− 34° 54′ 11.81″ S; − 56° 11′ 17.38″ W). Montevideo and its metropolitan area hosts > 1.5 M inhabitants, which represents ~50% of the country’s population. More than 90% of houses in this area are connected to the municipal sewage system, which collects wastewater and filters most macroscopic particles before its delivery 5 km away in the estuary. However, the western region of the city is not yet fully connected and their wastewater is poured directly on the shore or urban creeks. Previously, we used metagenomics to characterize the dissemination of antimicrobial resistance at 12 beaches and 8 sewage pipes sampled in August 2016 [11]. We now expanded this metagenomic study by including 22 samples collected in November 2017 that represent previous and new sampling points and environments across the city (Additional file 1: Table S1). In parallel, we performed an epidemiological investigation and whole-genome characterization of a multi-drug resistant outbreak that lasted between March and November 2017 (9 months) in one of the main public hospitals in the city, serving approximately half of the city’s population.

### Urban environments are differentially impacted by human-derived microbiota

**Fig. 1:**
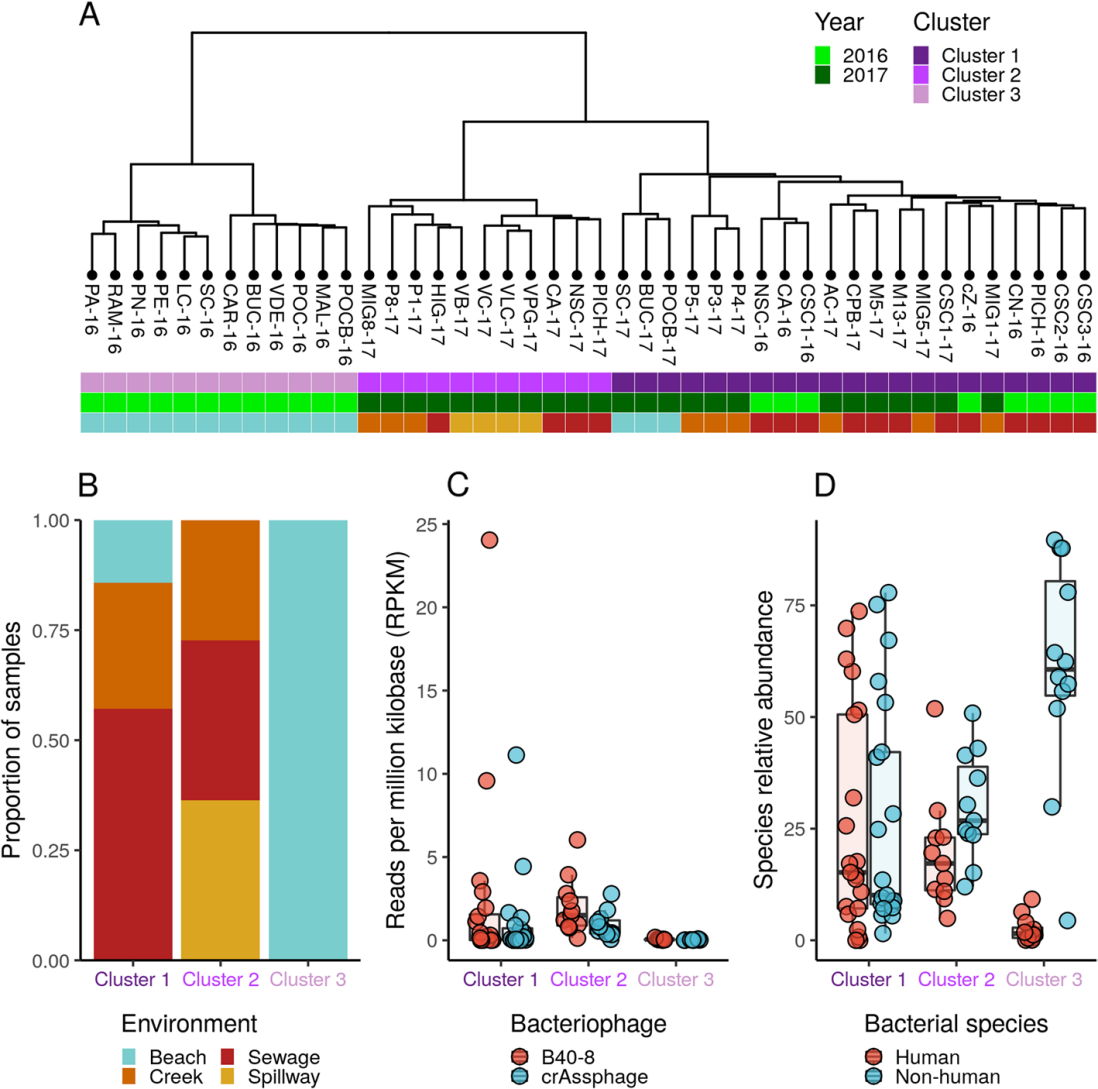
Dissemination of human bacteria in different niches of urban water environments. **A** Clustering analysis based on k-mers frequency from urban water metagenomes (*n* = 44). Samples are colored according to their belonging to each main cluster (clusters 1, 2, and 3), sampling year, and environment type according to their collection site (beach, sewage, creek, or spillway). **B** Barplot showing the proportion of samples belonging to each metagenomic cluster stratified by type of environment. **C** Metagenomic quantification (measured as reads per kilobase-million, RPKM) of human-gut-microbiota-associated bacteriophages B40-8 and crAssphage in samples belonging to each cluster. **D** Metagenomic quantification (measured as relative abundances) of bacterial species that are associated or not associated to the human gut microbiota in samples belonging to each cluster

First, we used a taxonomy-free clustering analysis based on shared *k-mer* counts between all metagenomic samples (*n* = 44). This revealed the presence of three distinct clusters. Cluster I is mostly dominated by sewage samples collected in 2016 and 2017. Cluster II is composed of samples from sewage pipes, spillways, and creeks collected in 2017. Cluster III contains beach samples collected in 2016 (Fig. 1A). Interestingly, the three clusters notably differed in their environmental composition and their exposure to human action: from expectedly less-impacted beaches, to mid-impacted urban creeks or spillways to more impacted environments where sewage water is directly poured (Fig. 1B).

Then, we evaluated the degree of human impact in these sites by measuring the abundance of the *Bacteroides* bacteriophages B40-8 and crAssphage, which have been previously shown to be suitable indicators of human pollution in the environment [12, 13]. Notably, the relative abundance of these phages gradually decreased from clusters defined by high to less impacted environments (Fig. 1C). We further tested this trend by measuring the relative abundance of bacterial species known to be part or not of the human microbiome (Fig. 1D). Indeed, the same gradual decrease of human-associated bacteria was clear from highly impacted to less impacted environments, while the opposite is observed for non-human bacteria. Together, these results uncover a defined structuring of microbial communities in differentially impacted urban environments that is driven by dissemination of human-associated bacteria.

### Human bacteria drives environmental dissemination of antimicrobial resistance

**Fig. 2:**
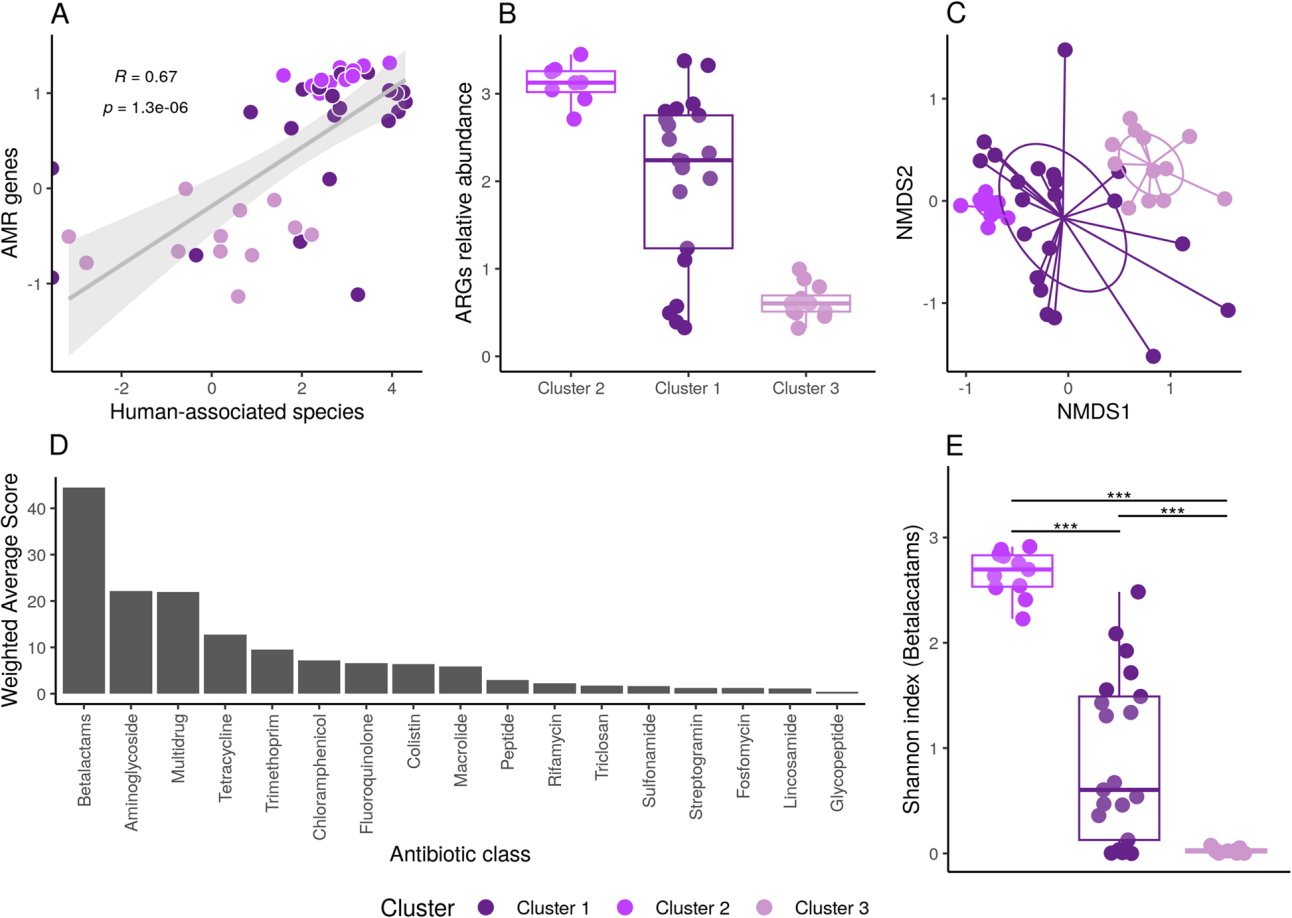
Dissemination of antimicrobial resistance (AMR) genes correlates with the presence of human bacteria. **A** Linear regression showing a positive and significant correlation (R = 0.67, *p*-value = 1.3e−6) between the relative abundance of human-associated bacterial species and the abundance of AMR genes. **B** Boxplots showing the relative abundance of ARGs in the three metagenomic clusters. **C** Non-metric multidimensional scaling (NMDS) analysis based on the abundance of AMR genes in all samples. Samples are colored by clusters. Variation in the horizontal axis (NMDS1) explains the separation of samples according to clusters based on their AMR gene repertory and abundance. **D** Barplot ranking antibiotic resistance gene classes that mostly contribute to the separation between samples observed in NMDS1 of panel B. Resistance to beta-lactams is the most contributing feature to separation of samples in the three predefined clusters. **E** Boxplot showing the alpha diversity (measured as the Shannon index) of beta-lactam resistance genes according to clusters. Level of statistical significance is shown as asterisks (*p* < 0.01)

We then aimed to investigate whether anthropogenic impact given by differential dissemination of human-associated bacteria in the urban environment could result in a higher burden of antimicrobial resistant genes (ARGs). We observed a positive and statistically significant correlation (*p* < 0.001; correlation coefficient = 0.67) between the relative abundance of human-associated bacteria and the overall abundance of ARGs in each sample (Fig. 2A). A higher abundance of AMR genes was observed in samples from clusters I and II than in cluster III (Fig. 2B). No significant correlation was observed when comparing with non-human-associated bacteria (*p* = 0.2; correlation coefficient = − 0.2) (Additional file 2: Figure S1). To further evaluate this, we used a non-metric multidimensional scaling (NMDS) analysis showing that ARGs presence/absence patterns in each sample recapitulate previously-defined clusters based on the whole microbial community (Fig. 2C). Based on this result, we sought to answer which discrete ARG classes were supporting the observed difference between clusters. We used the weighted average scores of each antibiotic class on the first dimension of the NMDS analysis (Fig. 2D) to show that resistance to beta-lactams was the major contributor to the observed differences. This was also observable by the statistically significant differences (*p* < 0.01) found between clusters when measuring the diversity of beta-lactam resistance genes (Fig. 2E). Together, these results provide evidence that human-associated bacteria may be a major driver to disseminate antimicrobial resistance in the urban environment, showcasing beta-lactam resistance as the most relevant antibiotic class distinguishing highly from low impacted sites.

### Concomitant increase of beta-lactam and carbapenem resistance in the urban and hospital environments

**Fig. 3:**
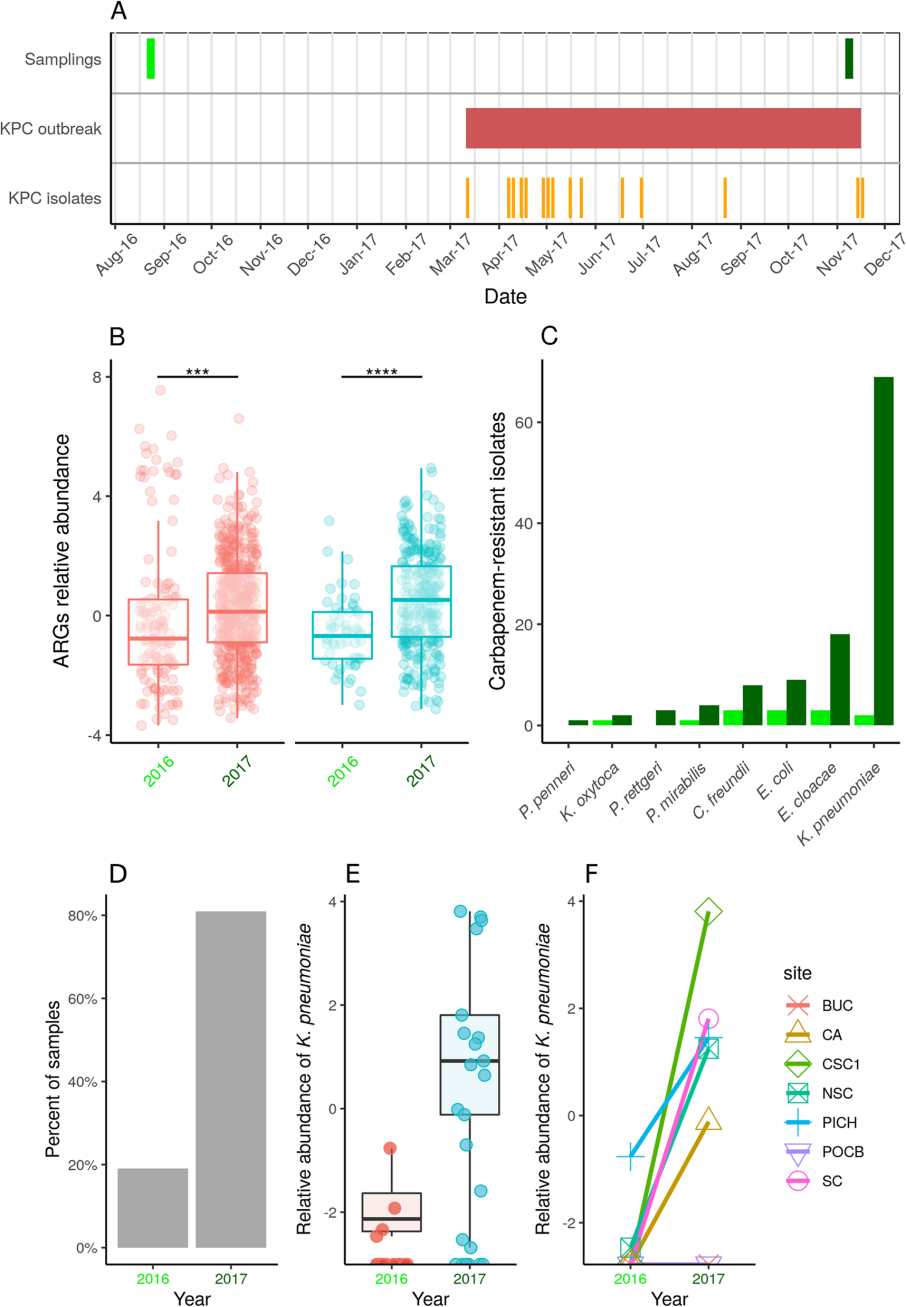
Relationship between beta-lactam and carbapenem resistance in the urban and hospital environment. **A** Timeline showing relevant collection dates. The top strip shows the dates when water samples were collected from the urban environment (August 2016 and November 2017). The strip in the middle shows the spanning time period of an outbreak registered in one of the main hospitals in the city caused by a carbapenemase-producing *Klebsiella pneumoniae* strain (KPC). The bottom strip shows the time when each *K. pneumoniae* isolate (*n* = 15) causing the outbreak was reported. **B** Boxplots showing the relative abundance of genes conferring resistance to beta-lactams (in red) in urban water samples collected in 2016 or 2017. The same is shown for carbapenem-resistance genes (in blue). Level of statistical significance is shown as asterisks (*p* < 0.01). **C** Barplot showing the number of nosocomial infections caused by different carbapenem-resistant *Enterobacteriaceae* in 2016 and 2017, at the same hospital where the KPC outbreak was reported. **D** Barplot showing the percentage of metagenomic samples collected in 2016 or 2017 that resulted positive for *K. pneumoniae*. **E** Relative abundance (logarithmic scale) of *K. pneumoniae* in all metagenomic samples collected in 2016 or 2017. **F** Relative abundance (logarithmic scale) of *K. pneumoniae* in paired metagenomic samples collected from the same sites in 2016 or 2017

Resistance to beta-lactam antibiotics (and carbapenems in particular) is one of the most common and problematic characteristics of hospital-acquired infections caused by multidrug resistant enterobacteria. Then, to contextualize our results, we evaluated the abundance of beta-lactam and carbapenem resistance genes in the urban environment and compared this to epidemiological data from nosocomial carbapenem-resistant infections in a 1-year period (2016–2017) that included a carbapenemase-producing *Klebsiella pneumoniae* outbreak consisting of 16 cases (Fig. 3A). The relative abundance for both beta-lactam and carbapenem resistance genes significantly increased (*p* < 0.01) in the urban environment between years (Fig. 3B). This was in line with a dramatic increase in nosocomial infections caused by carbapenem-resistant enterobacteria reported between 2016 and 2017, with *K. pneumoniae* as the top species (outbreak cases represented 25% of reported K. pneumoniae cases in 2017) (Fig. 3C). Also, when looking at the presence of *K. pneumoniae* in the urban environment, we observed a marked difference between these years, with 20% of *K. pneumoniae*-positive samples in 2016 to 80% of positive samples in 2017 (Fig. 3D). The same trend was detected when looking at the relative abundance of this species between 2016 and 2017 (Fig. 3E). Additionally, we compared paired samples collected from the same sampling sites in both years, confirming the previously observed increase of *K. pneumoniae* (Fig. 3F). In summary, these results show a concomitant increase in carbapenem resistance in the urban and hospital environments of the same city throughout a year, suggesting an important role of hospitalized patients’ microbiota in the dissemination of antimicrobial resistance to the urban environment.

### Genome-resolved metagenomics reveals environmental dissemination of nosocomial *K. pneumoniae* ST-11 clones

The marked increase in *K. pneumoniae* motivated us to determine whether the same clone was indeed being shared between hospital and urban settings. So, we first characterized the hospital-associated carbapenem-producing *K. pneumoniae* by generating complete whole-genome sequences for the 16 outbreak strains (Additional file 1: Table S2). Comparative analysis of these genomes confirmed epidemiological findings revealing their clonality, as they all belonged to the epidemic and multidrug-resistance associated sequence type 11 (ST-11), carried the siderophore yersiniabactin genotype 183 (in a genomic island of type ybt 9; ICEKp3), the capsular type KL64, and the O-locus type O2v1. The chromosome also encoded mutations in the *mgrB* gene conferring resistance to colistin and *gyrA* mutations conferring resistance to fluoroquinolones. Along with other mobile genetic elements, all genomes carried a ~146-kb megaplasmid (hereinafter referred as pKPC-146) encoding resistance to multiple antibiotics, including a type I integron carrying the *bla*_KPC-2_ carbapenem resistance gene (Additional file 2: Figure S2).

**Fig. 4:**
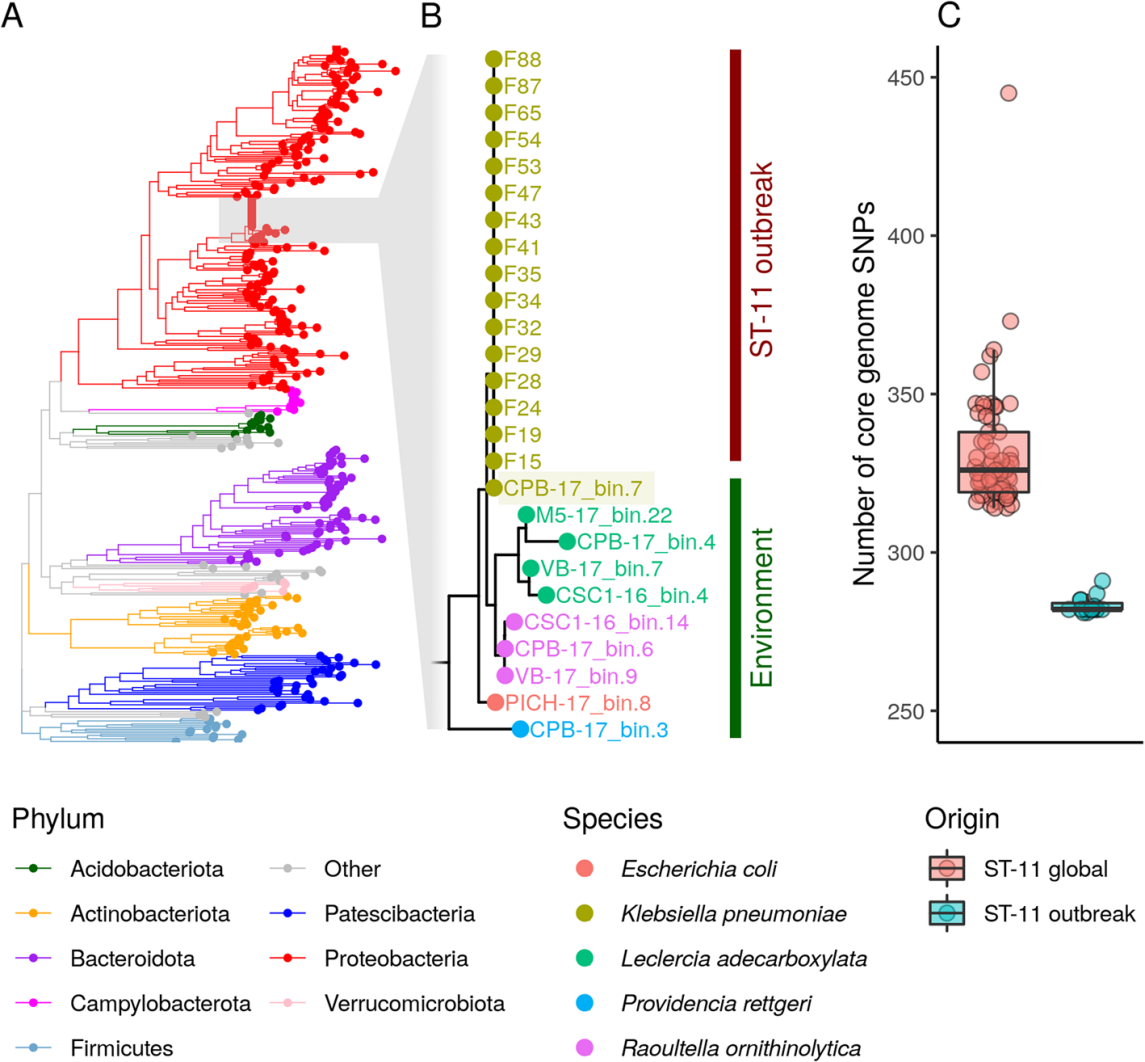
Genome-level analysis reveals transmission between hospital and urban environments. **A** Phylogenetic tree based on the concatenation of 120 conserved prokaryotic marker genes including 383 high- and medium-quality MAGs recovered from the urban environment and 15 high-resolution *K. pneumoniae* genomes sequenced from hospital-associated infections (ST-11 outbreak). Tree branches are colored according to taxonomy (phylum level). **B** Zoom-in the monophyletic clade corresponding to family Enterobacteriaceae. Tips are colored according to species and branches are grouped according to sample origin (ST-11 outbreak or environment). *K. pneumoniae* MAG recovered from sample CPB-17 (bin.7) is highlighted to show its phylogenetic association with *K. pneumoniae* nosocomial isolates recovered from the outbreak. **C** Boxplot showing the number of SNPs separating CBP-17_bin.7 from outbreak-associated *K. pneumoniae* ST-11 genomes or a global collection of non-outbreak *K. pneumoniae* ST-11 genomes

To perform a genome-level comparison to the outbreak strains, we generated 383 high- and mid-quality metagenome-assembled genomes (MAGs) from our urban metagenomes. Then, we used a phylogenomic approach to place the 16 *K. pneumoniae* ST-11 outbreak genomes in this context (Fig. 4A). As expected, the outbreak genomes clustered in a monophyletic clade along with environmental MAGs classified as enterobacterial species like *Escherichia coli*, *Leclercia adecarboxylata*, *Providencia rettgeri*, and *Raoultella ornithinolytica*. Interestingly, one MAG recovered from site CPB collected in 2017 was classified as *K. pneumoniae* and clustered with the outbreak genomes. MLST analysis of this MAG revealed it belongs to the ST-11 genotype (Fig. 4B) and has the same genomic markers as the outbreak strain (Additional file 1: Table S3). To further confirm the relatedness of this MAG with the outbreak-causing *K. pneumoniae* strain, we measured the number of SNPs distinguishing this MAG from the outbreak genomes or from a global collection of ST-11 genomes. This analysis revealed that the ST-11 MAG is in average ~70 SNPs closer to the outbreak genomes than to other ST-11 strains reported globally (Fig. 4C). In summary, these results support the presence of the same hospital-associated *K. pneumoniae* ST-11 clone in the urban environment after but not before the outbreak was reported in the hospital, highlighting the impact of hospital-derived fecal wastes in the dissemination of human pathogens and antimicrobial resistance in the urban environment.

### Geospatial dissemination of carbapenem-resistance inversely correlates with infrastructure of the sewage system

**Fig. 5:**
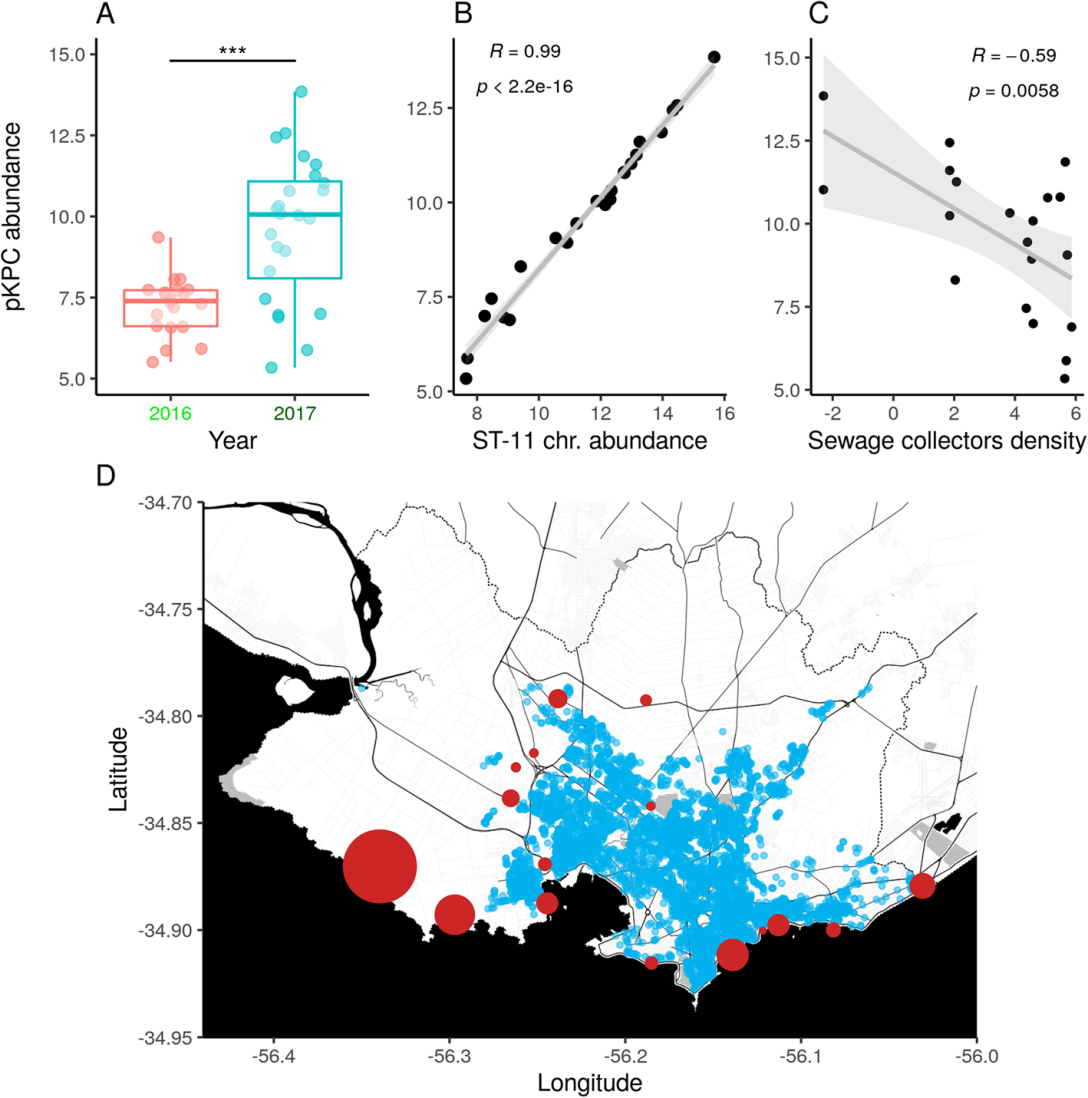
Geospatial dissemination of outbreak carbapenem-resistance plasmid depends on urban sewage infrastructure. **A** Boxplot showing a statistically significant (*p* < 0.01) increase in the abundance of the outbreak-associated carbapenem-resistance plasmid in urban metagenomes between 2016 and 2017. **B** Linear regression showing a positive and statistically significant correlation between the abundance of the outbreak-associated carbapenem-resistance plasmid and the *K. pneumoniae* ST-11 chromosome in urban metagenomes. **C** Linear regression showing a statistically significant and negative correlation between the abundance of the outbreak-associated carbapenem-resistance plasmid in each urban sampling point and the surrounding sewage infrastructure as measured as the density of wastewater collectors(pipes/km2). **D** Geospatial distribution of sewage collectors (blue dots) and sampling points (red circles) across the city of Montevideo. Size of red circles is proportional to the abundance of the outbreak-associated carbapenem-resistance plasmid

To better characterize the impact of human fecal contamination on the dissemination of antimicrobial resistance, we further investigated the geospatial distribution and abundance of the outbreak-associated carbapenem-resistance megaplasmid pKPC-146 in the urban environment (Fig. 5A). Our data showed that the relative abundance of pKPC-146 was significantly increased (*p* < 0.01) between 2016 and 2017. Additionally, the relative abundance of this plasmid was highly correlated (*p* < 0.01, correlation coefficient = 0.99) with the abundance of the *K. pneumoniae* ST-11 chromosome in the environment (Fig. 5B). Then, we aimed to determine if the degree of contamination with this multidrug resistant plasmid depended on the sewage system infrastructure. We hypothesized that urban areas with better sewage infrastructure more efficiently remove microbiological risks from the environment than less connected areas. Accordingly, we measured the abundance of the pKPC-146 plasmid in each sample from 2017 and defined the sewage infrastructure as the density of sewage collector pipes in a radius of 1.5 km from each sampling point. Indeed, we found a significant and negative correlation (*p* < 0.01; correlation coefficient = − 0.58) between abundance of pKPC-146 and sewage infrastructure (Fig. 5C). This is particularly evident in the western part of Montevideo when plotting the geospatial distribution of sewage collector pipes and points where pKPC-146 abundance was evaluated (Fig. 5D). These results provide evidence that the urban built-environment (infrastructure of sewage pipes in this case) can significantly affect the exposure to microbiological risks like highly resistant pathogens.

## Discussion

Our analyses indicate that different ecological niches within the sewage system and urban watercourses of a city enclose discrete microbial communities and that the main driver of these differences is the dissemination of bacteria associated with the human microbiome. Considering that the structure and composition of the human microbiome associates with diet, ethnicity, and geographic location [14, 15], the development of local-scale studies to characterize microorganisms in urban waters are particularly relevant to understand the local microbial landscape and AMR dissemination patterns. Such knowledge will enable scientists, clinicians, and policy-makers to understand how the activity of a city’s population and its infrastructure can impact health and well-being through changes in the environmental microbiome at a local scale.

Hospitals have been spotted as important reservoirs of AMR bacteria [3]. This issue is aggravated in developing countries where no primary treatment is in place to treat hospital wastewater before discharging it to the urban environment [16]. Other authors have shown that even though hospital wastewater had specific AMR signatures, this was erased when mixing with the urban wastewater due to the dilution rate [17]. Our study builds on this previous work by incorporating an extra layer of genomic information, since our genome-resolved metagenomic analysis uncovered the transmission of an outbreak-causing, multidrug resistant clone from the hospital to the city environment at the strain-level resolution. This is especially relevant because many enterobacterial species that encompass human pathogens have complex population structures, defined by environmental non-pathogenic lineages or high-risk, epidemic lineages. Hence, the capacity to detect and differentiate commensal from high-risk AMR strains belonging to the same species becomes imperative to leverage urban metagenomics as a high-resolution epidemiological surveillance tool.

This is important for *K. pneumoniae*, a recognized human pathogen that can also survive in diverse ecological niches and free-living conditions. Despite *K. pneumoniae* having a complex and large population structure, most infections are caused by a subset of global problem clones [4]. This is the case of the ST-11 clone, which has been shown to have prolonged survival on abiotic surfaces from the hospital built-environment with respect to other *K. pneumoniae* lineages [18]. The fact that ST-11 was the unique *K. pneumoniae* genome we could recover from the urban environment might indicate either an increased capacity of ST-11 to disseminate from the hospital to the urban environment or a major influence of the outbreak length in the environmental dissemination of this strain. This is particularly relevant considering that gut colonization with nosocomial enterobacteria, including *K. pneumoniae*, can exceed 12 months [19]. ST-11 is also one of the dominant extended-spectrum beta-lactamase and carbapenemase producing clones worldwide. Accordingly, its dissemination in the urban environment is coherent with the observed increase in beta-lactamic and carbapenem resistance genes in sewage waters through time and with retrospective data showing that some beta-lactamic represented over 70% of prescription in Uruguay in 2014 [20].

Importantly, the urban sewage sampling point from where we recovered a ST-11 MAG was about 20 km apart from the hospital where the outbreak was detected, suggesting the potential of outbreak strains to travel considerably long distances through the sewage system. This highlights the importance of carrying out more in-depth analyses to track the dissemination of AMR and epidemic bacterial clones through time and space in the urban environment. Anyway, our results uncover an important role of urban environments in the rise of extended-spectrum-beta-lactamase and carbapenemaase-producing microorganisms, which are associated with increased infection morbidity and mortality [21, 22].

Importantly, as resistance to beta-lactams and carbapenems is mainly coded in a wide diversity of plasmids, the discharge of human and hospital-associated strains into the environment generates favorable conditions for the transmission and dissemination of plasmids conferring resistance to these last-line antibiotics [23]. Overall, this reinforces the need for one-health approaches that integrate the surveillance of mobile elements to tackle the problem of antimicrobial resistance. Indeed, using this approach, we uncovered that the uneven sewage infrastructure across a defined urban area influenced the extensive spread of a novel, hospital-associated, carbapenem-resistant plasmid. This constitutes a major problem for public health given the release of sewage waters from deteriorating pipes or through sewer overflows that can expose the population to potentially harmful bacteria and increase the risk of enteric infections [24]. In the future, we think it crucial to integratively analyze the circulation of pathogenic bacteria and AMR measured directly from the sewage waters with urbanism and infrastructure planning and epidemiological data collected from the population. For example, availability of systematic and retrospective data on antibiotic use and prescription is central to fully understanding or even predicting AMR dynamics in the environment and the population.

## Conclusions

Our results evidence the need of urgent global actions to tackle the problem of AMR dissemination for a one-health perspective. Emphasis should be put on the role of urban human populations as a reservoir and vehicle for AMR emergence and spread through wastewater systems. In particular, the relationship between hospital effluents with pathogen circulation and survival in the environment represents a threat for public health which requires the development and implementation of improved hospital wastewater treatment protocols. Indeed, we provide evidence that the occurrence of prolonged nosocomial outbreaks caused by epidemic and multidrug resistant pathogens like *K. pneumoniae* can modify the urban microbiome and that the extent of this can be influenced by the built-environment infrastructure. Finally, the deployment of longitudinal and longer term wastewater surveillance programs using metagenomics represent a promising tool that not only can improve our understanding of pathogen dynamics and other microbiome-related diseases, but also provide useful information for infection control, public health policies, and urban design.

## Methods

### Environmental sample collection and metagenomic data generation

In November 2017, twenty four environmental water samples were collected as previously described [11]. These locations were selectively chosen to represent beaches, creeks, spillways, and sewage collector pipes within the Montevideo city metropolitan area. A brief description of the location, date of sampling and associated metadata is summarized in Additional file 1: Table S1. All samples were collected in sterile 200 mL bottles and kept on ice. Then, samples were processed as previously described [11]. Briefly, they were centrifuged at 10,000×g for 15 min, the supernatants were discarded, and pellets were processed using the FastDNA™ Spin Kit (MP Biomedicals) following the manufacturer’s protocol. DNA was sequenced using a shotgun metagenomic approach using paired end (2 x 150 bp) reads with an Illumina HiSeq4000 platform. Additionally, shotgun metagenomic sequencing data generated from 20 samples collected in August 2016 and previously published from our group [11] were retrieved for further analysis (PRJNA515946). The combined dataset consisted of 44 metagenomic samples of 42.3M paired-end reads on average (min: 23M, max: 64.8M).

### Bacterial isolation, characterization and whole-genome sequencing

Bacterial colonies were obtained from blood samples and identified at the species level using the VITEK 2 y VITEK MS system (bioMérieux), confirming the presence of *Klebsiella pneumoniae*. Antimicrobial susceptibility was determined by VITEK 2, disk diffusion, and E-test according to CLSI 2019 cutoffs. Detection of carbapenemase production was performed using the GeneXpert instrument. Susceptibility to colistin was studied with the colistin broth disk elution test. Genomic DNA was purified with Purelink Genomic DNA kit (Invitrogen). DNA was sequenced using paired end (2 × 150 bp) reads with an Illumina HiSeq4000 platform. Also, DNA was fragmented using a G-tube (Covaris) to approximately 8-Kb fragment distribution, according to manufacturer’s instructions. Then, long-read sequencing libraries were prepared using the Native barcoding genomic DNA by Ligation (SQK-LSK109) protocol (Oxford Nanopore Technology, ONT) and loaded into a R9.4.1 FLO-MIN106D flow cell. Basecalling was performed using Guppy 3.2.9 installed on a MinIT device. Demultiplexing and adapter sequence removal was performed with Porechop v0.2.3 (https://github.com/rrwick/Porechop). Reads were filtered using NanoFilt v2.3.0 (q score > 8, length > 500).

### Metagenomic assembly, binning and taxonomic classification

Metagenomic reads from each analyzed sample were assembled using metaSPAdes [25] with default parameters. Then, metagenome-assembled genomes (MAGs) were reconstructed using metaWRAP binning, refinement, and reassembly modules [26]. CheckM [27] was used to evaluate MAGs completion and contamination. Only mid-quality (contamination < 5%; completion >= 50%) and high-quality (contamination < 5%; completion > = 90%) MAGs were used for downstream analyses. The classification workflow from the GTDB-tk [28] was used to obtain MAGs taxonomic classification.

### Assembly and characterization of *K. pneumoniae* genomes

Hybrid assemblies combining Illumina short-read and ONT long-read data were generated using Unicycler [29] with default parameters and visualized using Bandage [30]. Sequence type was initially determined using MLSTar [31]. In depth genomic characterization including detection of virulence genes and AMR genes was performed with Kleborate [32]. Plasmids were classified using PlasmidFinder [33].

### Comparative metagenomic analyses

SimkaMin [34] was used to clusterize metagenomic samples according to their overall genetic relatedness using read k-mer distances. Relative abundance of bacterial species was determined using MetaPhlAn2 [35]. Definitions of bacterial species associated or not to the human microbiome were obtained from the Microbe Directory, a manually-curated database of microbes’ characteristics [36]. Estimation of bacteriophage abundances was performed by mapping metagenomic reads against crAssphage and B40-8 reference genomes using bowtie2 [37]. Reads per million kilobase (RPKM) values were calculated in R [38]. Relative abundance of ARGs in metagenomic samples was estimated using ShortBRED [39] and the CARD database [40]. Sample clustering based on ARGs relative abundance was determined using a non-metric multidimensional scaling (NMDS) approach implemented in the vegan package [41]. Weighted average scores for dimension 1 were extracted for each antibiotic class and ranked to determine the top contributing antibiotic classes to the observed clustering. For beta-lactamic ARGs, alpha diversity was (Shannon index) calculated using the vegan package [41].

### Phylogenetic analyses

Phylogenetic reconstruction was performed using 383 mid- and high-quality MAGs and the 16 *K. pneumoniae* outbreak genomes, based on the approach implemented by the GTDB-tk [28] which search, concatenate and align 120 conserved bacterial marker genes. Plots were generated with the ggtree package [42]. Core genome SNPs were calculated using Snippy (https://github.com/tseemann/snippy) by comparing the CPB-17 MAG against the 16 *K. pneumoniae* outbreak genomes or to a collection of 873 global *K. pneumoniae* ST-11 genomes obtained from the PATRIC database [43].

### Geospatial analyses

The prevalence of the pKPC-146 plasmid in the different sampling locations was calculated by mapping metagenomic reads against the complete plasmid genome using bowtie2 [37], and then reads per million kilobase (RPKM) values were calculated in R [38]. The geospatial distribution of sewage pipes in the city of Montevideo was obtained from the Spatial Data Infrastructure (https://www.gub.uy/infraestructura-datos-espaciales/) system of Uruguay. Density of sewage built infrastructure was calculated as the number of sewage pipes in a radius of 2 km around each sampling point. Visualizations were generated with the ggplot2 [44] and ggmap [45] packages.

## Supplementary Information

**Additional file 1: Supplementary Table S1.** Metadata of analyzed samples. **Supplementary Table S2.** Antimicrobial resistance phenotypes of K. pneumoniae ST-11 outbreak strains. **Supplementary Table S3.** Genomic characterization of K. pneumoniae ST-11 isolates and MAG.

**Additional file 2: Supplementary Figure S1.** Analysis of ARGs and non-human species. Lack of significant linear correlation between the relative abundance of ARGs and non-human species. Dots are colored according to the three metagenomic clusters. **Supplementary Figure S2.** Schematic representation of the pKPC-146 plasmid. Circular representation of the pKPC-146 plasmid with annotated genes. Genes are colored according to their functions: plasmid conjugation (skyblue), hypothetical (gray), antibiotic resistance (green), mercuric resistance (yellow), other functions (purple), transposons (pink). The KPC carbapenemase gene is highlighted in red.

## Data Availability

The datasets generated and/or analyzed during the current study are available in the FigShare repository, 10.6084/m9.figshare.20304078.v2. Sequencing reads generated in this study have been deposited in the NCBI repository, project identifier PRJNA857878.

## Abbreviations

KPC: *Klebsiella pneumoniae* carbapenemase
AMR: Antimicrobial resistance
WHO: World Health Organization
ARGs: Antimicrobial resistance genes
NMDS: Non-metric multidimensional scaling
ST-11: Sequence type 11
MAGs: Metagenome-assembled genomes
MLST: Multilocus sequence typing
ONT: Oxford Nanopore Technologies
