# Supplementary material for: Human microbiota drives hospital-associated antimicrobial resistance dissemination in the urban environment and mirrors patient case rates
Source: Microbiome. 2022 Dec 2;10:208. doi: 10.1186/s40168-022-01407-8 (PMC9715416; doi:10.1186/s40168-022-01407-8)
Supplement: Supplementary file 3 — Additional file 2: Supplementary Figure S1. Analysis of ARGs and non-human species. Lack of significant linear correlation between the relative abundance of ARGs and non-human species. Dots are colored according to the three metagenomic clusters. Supplementary Figure S2. Schematic representation of the pKPC-146 plasmid. Circular representation of the pKPC-146 plasmid with annotated genes. Genes are colored according to their functions: plasmid conjugation (skyblue), hypothetical (gray), antibiotic resistance (green), mercuric resistance (yellow), other functions (purple), transposons (pink). The KPC carbapenemase gene is highlighted in red. [file 40168_2022_1407_MOESM2_ESM.pdf]

Supplementary Figures

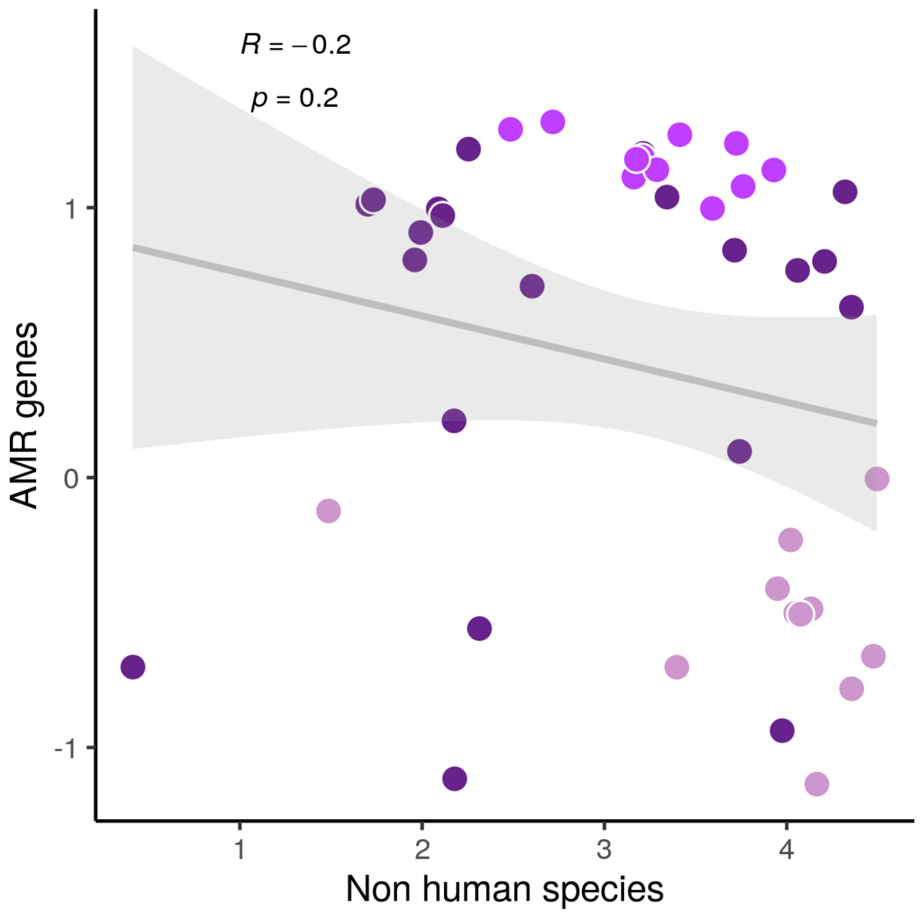

Supplementary Figure S1. Analysis of ARGs and non-human species. Lack of significant linear correlation between the relative abundance of ARGs and non-human species. Dots are colored according to the three metagenomic clusters.

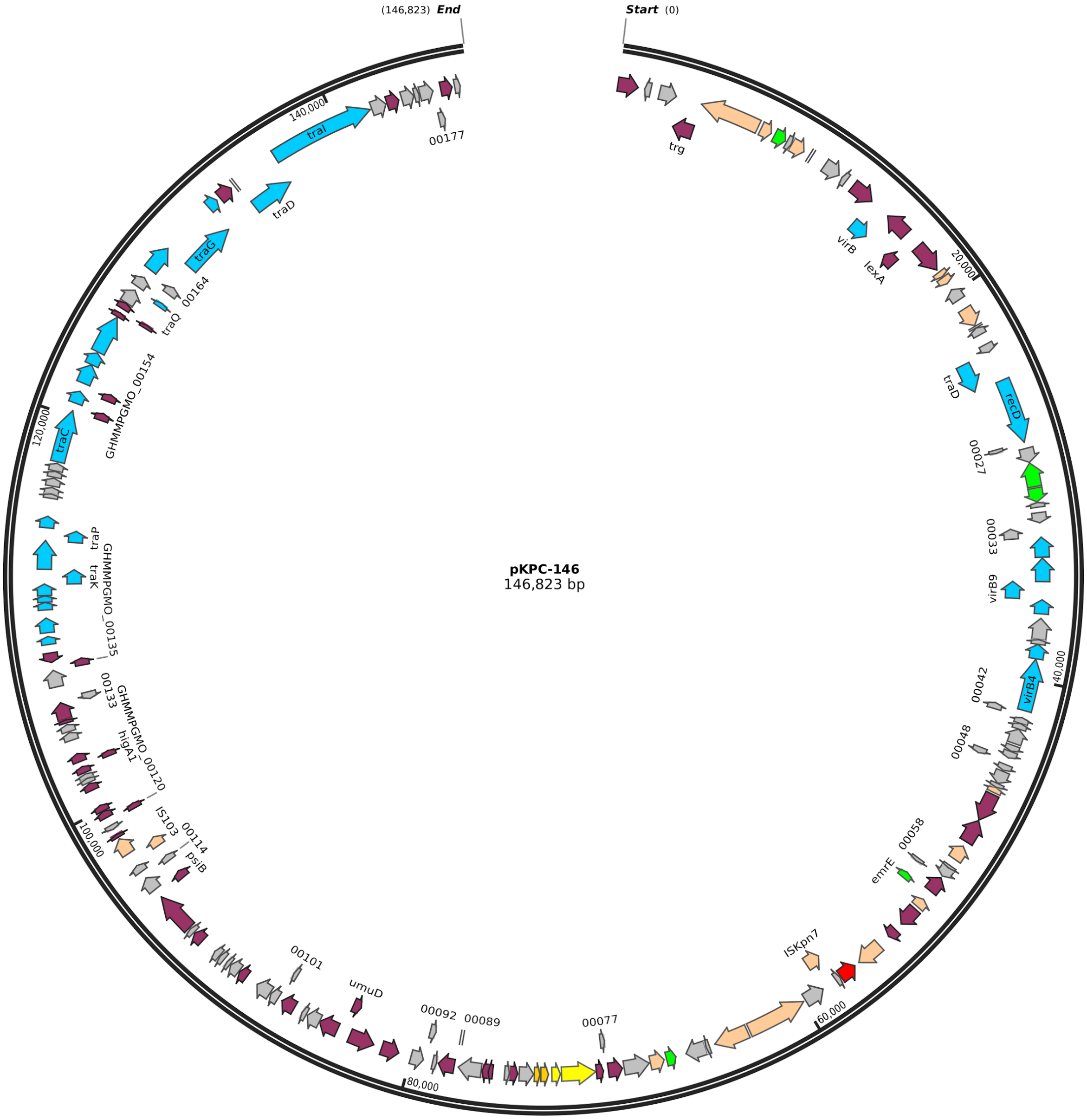

Supplementary Figure S2. Schematic representation of the pKPC-146 plasmid. Circular representation of the pKPC-146 plasmid with annotated genes. Genes are colored according to their functions: plasmid conjugation (skyblue), hypothetical (gray), antibiotic resistance (green), mercuric resistance (yellow), other functions (purple), transposons (pink). The KPC carbapenemase gene is highlighted in red.
